# Protective Effects of Omega-3 Fatty Acids in Cancer-Related Complications

**DOI:** 10.3390/nu11050945

**Published:** 2019-04-26

**Authors:** Raquel D. S. Freitas, Maria M. Campos

**Affiliations:** 1 Centro de Pesquisa em Toxicologia e Farmacologia, Escola de Ciências da Saúde, PUCRS, Porto Alegre 90619-900, RS, Brazil; raqueldalsasso@gmail.com; 2 Programa de Pós-graduação em Medicina e Ciências da Saúde, Escola de Medicina, PUCRS, Porto Alegre 90619-900, RS, Brazil; 3 Programa de Pós-graduação em Odontologia, Escola de Ciências da Saúde, PUCRS, Porto Alegre 90619-900, RS, Brazil

**Keywords:** omega-3, cancer, nutrition, anorexia-cachexia syndrome, pain, depression, paraneoplastic syndromes

## Abstract

Omega-3 polyunsaturated fatty acids (PUFAs) are considered immunonutrients and are commonly used in the nutritional therapy of cancer patients due to their ample biological effects. Omega-3 PUFAs play essential roles in cell signaling and in the cell structure and fluidity of membranes. They participate in the resolution of inflammation and have anti-inflammatory and antinociceptive effects. Additionally, they can act as agonists of G protein-coupled receptors, namely, GPR40/FFA1 and GPR120/FFA4. Cancer patients undergo complications, such as anorexia-cachexia syndrome, pain, depression, and paraneoplastic syndromes. Interestingly, the 2017 European Society for Clinical Nutrition and Metabolism (ESPEN) guidelines for cancer patients only discuss the use of omega-3 PUFAs for cancer-cachexia treatment, leaving aside other cancer-related complications that could potentially be managed by omega-3 PUFA supplementation. This critical review aimed to discuss the effects and the possible underlying mechanisms of omega-3 PUFA supplementation in cancer-related complications. Data compilation in this critical review indicates that further investigation is still required to assess the factual benefits of omega-3 PUFA supplementation in cancer-associated illnesses. Nevertheless, preclinical evidence reveals that omega-3 PUFAs and their metabolites might modulate pivotal pathways underlying complications secondary to cancer, indicating that this is a promising field of knowledge to be explored.

## 1. Introduction

Bang and Dyerberg investigated the Greenland Eskimo diet in the 1970s in order to determine the reason for why this population had a low prevalence of cardiovascular diseases. The Eskimo diet was composed of seal and whale blubber, containing high protein and low carbohydrate levels, as well as the same amount of fat, when compared to the regular Danish diet [1,2]. The leading cause of the low prevalence of cardiovascular diseases had been attributed to the high dietary contents of omega-3 polyunsaturated fatty acids PUFAs) [3].

Omega-3 PUFAs are classified as essential because they cannot be synthesized by the organism; hence, the consumption of food rich in omega-3, such as fish from cold waters, nuts, and seed oils, is mandatory [4]. The beneficial effects of omega-3 PUFA consumption are likely related to its anti-inflammatory and pro-resolution effects, mainly due to the inhibition of nuclear factor kappa B (NF-κB) and the production of pro-resolution mediators, such as resolvins, protectins, and maresins [5,6]. More recently, two G protein-coupled receptors, called Free Fatty Acid Receptor 1 (FFA1) and Free Fatty Acid Receptor 4 (FFA4), were identified as molecular targets for omega-3 PUFAs [7,8]. When activated, these receptors can promote a number of effects, such as improving the insulin sensibility, inducing adipose tissue browning, promoting analgesia by the release of β-endorphin, controlling energy homeostasis, and diminishing food intake [9,10,11,12].

According to GLOBOCAN 2018, a project of the International Agency for Research on Cancer, 18.1 million new cases of cancer and 9.6 million cancer-related deaths worldwide were estimated for the year 2018. For 2020, 17 million new cases are estimated; 66% will live for nearly five years, and at least 40% will live for more than 10 years after diagnosis. Every year, 8.5 million people die from cancer [13]. Lung cancer is the most diagnosed type of cancer for both sexes and the leading cause of death. In males, lung cancer is also the most common and the first cause of death, followed by prostate and colorectal cancers. Among women, breast cancer is the leading type of cancer and the main cause of death, followed by colorectal and lung cancers [13].

As stated by the International Association for the Study of Pain, pain is an “unpleasant sensory and emotional experience, associated with actual or potential tissue damage or described in terms of such damage”, and neuropathic pain is the principal type of pain in cancer patients [14,15,16]. Cancer pain is the most common cancer-related complication, reported by approximately 90% of patients. Unfortunately, up to 50% of these patients are poorly treated for this condition. Pain in cancer patients occurs because of the tumor growth itself, metastasis development, or treatment-related adverse effects, such as chemotherapy-induced neurotoxicity. Pain in cancer survivors is also important because any change in this condition can indicate a recurrence of the tumor [14,15,17].

Another important cancer-associated complication is anorexia-cachexia syndrome, which affects up to 85% of cancer patients [18]. This condition is defined as a multifactorial syndrome with muscle atrophy, fat loss, and the progressive defeat of function, leading to a low quality of life, which cannot be reversed by conventional nutritional therapy [19]. Skeletal muscle atrophy and an increase of energy balance occur due to systemic inflammation and a reduced appetite. Particularly, systemic inflammation is the main mechanism for the development of proteolysis, lipolysis, insulin resistance, and a high resting energy expenditure in this condition [20]. Likewise, tumor-derived factors have similar roles as inflammatory cytokines regarding the catabolic effects. Additionally, cancer patients who develop cachexia lose their independence regarding daily chores, leading to a low quality of life [21,22].

The diagnosis of cancer can provoke stress and sadness, leading to major depressive disorder (MDD). However, MDD is not only explained by the emotional impact of the diagnosis. Pro-inflammatory cytokines related to cancer and/or treatment play a key role in cancer-related depression [23]. The prevalence of depression in cancer patients is around four times higher than in the general population, although it does not increase with the severity of the disease. On the other hand, depression is associated with a poor prognosis in cancer patients, mainly due to the low adherence to treatment, which is caused by a lack of family ties and social support, by a history of childhood trauma, and by adverse life experiences [24,25].

Paraneoplastic syndromes involve a wide variety of symptoms related to tumor presence but are not associated with development and malignancy, being a result of the tumor-induced release of hormones or peptides. Unlike the conditions cited above, in general, paraneoplastic syndromes only affect 8% of all cancer patients [26]. Interestingly, the presence of a paraneoplastic syndrome can lead to a cancer diagnosis. Paraneoplastic disorders can have a high mortality rate, but they are manageable and curable after cancer treatment. Due to the rarity of these cases, clinical evidence and guidelines to aid treatment are still lacking when compared to other cancer-related complications [26,27,28].

Considering that approximately 20% to 80% of cancer patients use dietary supplements after diagnosis [29], the severity of cancer-associated complications, and the beneficial effects of omega-3 PUFAs, the purpose of this critical review article is to discuss the possible mechanisms and effects of omega-3 PUFA supplementation in principal cancer-related disorders.

## 2. Omega-3 PUFAs and the Possible Mechanisms of Action in Cancer Complications

Omega-3 PUFAs are essential fatty acids, containing between 18 and 22 carbons, with the first double bond on the third carbon, counting from the omega end. Omega-3 PUFAs comprise three different active molecules: (i) α-linolenic acid (ALA; 18:3n-3), (ii) eicosapentaenoic acid (EPA; 20:5n-3), and (iii) docosahexaenoic acid (DHA; 22:6n-3). ALA is synthesized in plants and can be found in seeds, nuts, and plant oils. EPA and DHA are not synthesized by the organism and can only be found in the flesh of cold-water fish [30]. Interestingly, ALA can be converted to EPA and DHA by several reactions of elongation and desaturation, but these conversions produce small amounts of EPA and DHA in the organism [31].

The omega-6 arachidonic acid (AA; 20:4n-6) and linoleic acid (LA; 18:2n-6) are also essential fatty acids. Notably, both became major components of the cell membrane due to the increase of Western diets, rich in cereals and vegetable oils, containing excessive omega-6 PUFAs and leading to an undesired omega-6/omega-3 ratio of 20:1 [32]. The metabolic pathways of AA and LA share the same enzymes that convert ALA to EPA and DHA, indicating that there is competition between the pathways. In inflammatory processes, membrane phospholipids are cleaved by phospholipase A2 (PLA2) to release AA to the cytoplasm and initiate the production of highly inflammatory eicosanoids (such as prostaglandin E2 and leukotriene B4) by the action of cyclooxygenases and lipoxygenases. The membrane lipid composition modification from an omega-6 PUFA to omega-3 PUFA profile is very important because it increases the production of omega-3-derived mediators, such as thromboxane A3 and prostacyclin I3, which are weaker inducers of inflammation [33]. Supporting this mechanism, a systematic review and meta-analysis demonstrated that omega-3 PUFAs were able to reduce thromboxane B2 blood levels in subjects with a high risk of cardiovascular diseases, along with a decrease of leukotriene B4 in the neutrophils of unhealthy patients [34]. Regarding lymphocyte membranes, an in vitro and pilot clinical study evaluated the fatty acid composition of CD4+T cell membranes after EPA and DHA supplementation. The in vitro analysis showed that EPA or DHA incubation increased the membrane contents of omega-3 PUFAs. Additionally, the pilot clinical study from the same article evaluated the membrane composition of lymphocytes in elderly individuals after six weeks of omega-3 PUFA supplementation and observed a similar omega-3 PUFA-rich membrane [35]. Additionally, a review article demonstrated that EPA and DHA supplementation are often employed in the nutritional therapy of cancer patients and promotes beneficial effects during cancer treatment due to a membrane modulation [36]. On the other hand, an analysis of the fatty acid composition of the red blood cells of cancer patients showed that there was no difference between the omega-3 PUFAs contents in the membrane of cancer patients and healthy subjects, irrespective of their diet. Interestingly, the same cancer patients showed higher omega-6 PUFA contents and an increased desaturation activity, demonstrating a higher inflammatory profile [37].

The notion that an omega-3 PUFA-enriched membrane could be favorable for disease management was corroborated by the discovery of pro-resolution mediators of inflammation, derived from omega-3 PUFAs. Over the past decade, the identification of resolvins, protectins/neuroprotectins, and maresins was a milestone—currently, it is well-recognized that solving, rather than inhibiting, inflammation is quite an interesting approach for the treatment of a series of chronic illnesses such as cancer.

In acute inflammation, the production of prostaglandins by the action of cyclooxygenases-1 and -2 is essential for blood flow regulation and an increase of endothelial permeability. Additionally, the production of leukotrienes is required for leukocyte migration [38]. Notably, it was believed that all products of the inflammatory process, such as eicosanoids, prostanoids, cytokines, and chemokines, are diluted over time and that the inflammation process would be resolved [39]. Nevertheless, studies demonstrated that a group of lipid pro-resolution mediators, derived from arachidonic acid (AA), namely lipoxins, were crucial to stopping the pro-inflammatory signals, indicating that the resolution of inflammation is an active process [40]. Lipoxins can inhibit the entrance of new neutrophils and stimulate macrophages to clear apoptotic neutrophils [41]. Remarkably, omega-3 PUFAs are crucial for the generation of potent pro-resolution mediators, with similar actions to lipoxins, such as resolvins, protectins, neuroprotectins, and maresins. Resolvins are divided into the series E (RvE) and D (RvD), originating from EPA and DHA, respectively. As for protectins, neuroprotectins and maresins originate from DHA, but maresins are produced only by macrophages [42,43,44]. These mediators of resolution can decrease the leukocyte infiltration and reduce cellular debris, leading to the cessation of the inflammatory process [44]. Notably, they have been widely investigated, showing beneficial effects in a series of preclinical inflammation models. Regarding the effects of these pro-resolution mediators in cancer, RvD1, RvD2, and RvE1 were capable of reducing the debris-stimulated cancer progression by inducing macrophage phagocytosis and diminishing pro-inflammatory cytokines [45]. Likewise, DHA-derived pro-resolution mediators, such as neuroprotectin D1, maresin 1, and RvD1 and RvD5, displayed important analgesic effects in a mouse model of postoperative pain after bone fracture when administered after surgery. Nevertheless, the same study demonstrated that DHA administration before surgery partially reduced postoperative pain due to the conversion of DHA to pro-resolution mediators [46]. Concerning the effects of resolution mediators on depression, RvE1 and RvE2 intracerebroventricular (i.c.v.) administration significantly decreased lipopolysaccharide (LPS)-associated depressive behavior via the activation of the resolvin receptor ChemR23 according to the assessment of LPS-induced depression in a mouse model [47]. Similarly, a study of our group revealed the beneficial effects of RvD2 treatment in the depression-like behavior in a mouse model of fibromyalgia [48]. A critical review speculated that the resolution of inflammation is flawed in cancer-cachexia, suggesting that the induction of the resolution process would be beneficial for cancer-cachectic patients [49]. Surprisingly, there are no experimental or clinical studies investigating the effects of pro-resolution mediators in cancer cachexia.

Omega-3 PUFAs can activate G protein-coupled receptors, generating intracellular effects. Firstly, Briscoe et al. (2003) identified the FFA1 receptor, formerly known as the G-protein coupled receptor 40 (GPR40), as a free fatty acid receptor. It was observed that long-chain fatty acids could cause a concentration-dependent increase in intracellular calcium in human embryonic kidney (HEK293) cells expressing FFA1 [7]. The expression of the FFA1 receptor indicates that this receptor is an important molecular target for metabolism control, as observed in the gastrointestinal tract, pancreatic β-cells, and brain [50,51,52]. Regarding the effects of FFA1 in the metabolism, the activation of this receptor is associated with glucagon-like peptide-1 (GLP-1) and cholecystokinin release [53,54]. More recently, it was observed that the FFA1 receptor is expressed in the melanocortin system, specifically in the neuropeptide Y/Agouti-related peptide (NPY/AgRP) and proopiomelanocortin/cocaine- and amphetamine-regulated transcript (POMC/CART) neurons [55]. Interestingly, the FFA1 expression is upregulated in other tissues under pathological situations, such as periodontitis, which is associated with metabolic syndrome [56]. Regarding the antidiabetic effect, FFA1 has been widely investigated as a molecular target for diabetes, mainly due to the glucose-stimulated insulin secretion via protein kinase C/inositol triphosphate (PKC/IP_3_) activation and, consequently, intracellular calcium increase, inducing insulin release [57,58]. In virtue of this effect, TAK-975, a synthetic selective FFA1 agonist, was tested until phase II of clinical trials for diabetes management. Unfortunately, the clinical investigation had been interrupted because patients developed hepatoxicity and liver failure [59,60]. More recently, the role of the FFA1 receptor in the central nervous system has attracted interest. The activation of this receptor by DHA demonstrated analgesic effects in different experimental pain models [11,12,61,62]. As for FFA1 ligands, long-chain fatty acids are considered endogenous agonists, mainly DHA, but the studies demonstrated that oleic acid is also a potent FFA1 agonist [10,61,62,63].

After the identification of FFA1 as a free fatty acid receptor, FFA4, formerly known as G-protein-coupled receptor 120 (GPR120), was also identified as part of this new family of G-protein coupled receptors. On the subject of FFA4 ligands, EPA, ALA, and DHA are considered endogenous ligands, but the latter presents a lesser potency [64]. Similar to FFA1, FFA4 is also activated by long-chain fatty acids and also has metabolic functions [8,65]. Notably, the expression of this receptor can be induced by a fish oil-enriched diet and by the aerobic exercise of different organs [66,67]. Osteoclasts and osteoblasts also express FFA4, and in the presence of high levels of omega-3 fatty acids, it can promote bone formation and inhibit bone resorption [68]. The FFA4 receptor can be found in the taste buds, liver, adipose tissue, intestines, macrophages, and pancreas [69]. Interestingly, the human FFA4 receptor exists in two isoforms: long and short. The long has a 16-residue segment in the third intracellular loop that decouples the receptor from the G protein. However, both isoforms can activate β-arrestin-2, recruiting the transforming growth factor β-activated kinase 1(TAK1)-binding protein 2 (TAB2), which inhibits TAK1, leading to anti-inflammatory effects [65]. As for the main mechanism of action of FFA4, the activation of this receptor leads to G_q/11_ protein activation, stimulating IP_3_ and, consequently, increasing intracellular calcium concentration, resulting in hormone secretion. Regarding the effects of FFA4 in obesity, it was observed that FFA4 is localized in NPY-positive neurons, indicating that FFA4 activation by omega-3 PUFAs can decrease appetite, food reward, and anxiety-like behavior [70,71]. At last, the activation of FFA4 induces the browning of the adipose tissue (white adipocytes are transformed into beige adipocytes), indicating another mechanism against the development of obesity [9,72]. Thus, it is tempting to suggest that FFA4 activation by omega-3 PUFAs might interfere with cancer bone metastasis and cachexia, although further studies on this hypothesis are still required.

## 3. Omega-3 PUFAs as Part of Pharmaconutrition in Cancer Patients

The areas of immunonutrition and pharmaconutrition have emerged due to the impact of nutrients in the organism being greater than the nutrition itself. Nevertheless, pharmaconutrition, which is characterized by nutrient supplementation at pharmacological doses, seems to be more promising than immunonutrition, defined as a nutrient-enriched diet [73]. Malnourished cancer patients can display a diminished response to cancer therapy, increase in infections, an extension of the length of hospital stay, an augmentation of the risk of postoperative complications, and death [74,75]. Patients may experience mechanical and functional alterations, especially when the tumor is located in the gastrointestinal tract. Additionally, they can display adverse effects related to cancer treatment, such as nausea, vomiting, mucositis, xerostomia, and/or dysphagia [76]. Also, a high inflammatory state in cancer patients might be related to cancer complications, such as depression, cachexia, pain, and paraneoplastic syndromes [77,78]. Immunonutrition with omega-3 PUFAs, glutamine, arginine, and ribonucleotides is often prescribed to cancer patients and is believed to maintain immunocompetence during the treatment [79,80]. Conversely, other clinical randomized trials observed that immune-enhancing diets, when offered to cancer patients, failed to improve the immune response and were no different from standard diets [81,82,83]. Alternatively, pharmaconutrition is employed as a nutrient supplementation during cancer treatment in order to diminish treatment-related complications. Currently, omega-3 PUFAs can be considered as pharmaconutrients, acting as receptor agonists, modulating molecular pathways, reducing the inflammatory response, increasing the chemotherapy efficacy, and consequently improving the overall survival of cancer patients [84,85,86]. Curiously, low contents of omega-3 PUFAs in the mammary region seem to contribute to breast cancer multifocality, indicating that omega-3 PUFA supplementation is important for cancer management and prevention [87]. Therefore, omega-3 PUFA-based pharmaconutrition is likely useful for handling cancer-related outcomes.

## 4. Cancer-Related Pain

Most cancer patients experience different types of pain associated with the disease. Cancer patients often report intense pain, leading to a lower performance status [88]. Pain might be related to tumor localization, but it can also arise due to chemotherapy treatment and/or surgery [89]. Notably, cancer pain comprises inflammatory and neuropathic mechanisms in virtue of tumor mass development [90]. Signaling molecules that are released by the environment are responsible for tissue remodeling and for tumor invasion and metastasis. These molecules can be pro-inflammatory cytokines, chemokines, and growth factors, which are released by cells in order to modulate tumor growth [91]. Additionally, chemo- and radiotherapy induce toxicity and inflammation, evoking painful symptoms, decreasing patients’ quality of life and, consequently, diminishing the treatment adherence [92].

Peculiarly, we were not able to find any clinical or experimental studies on omega-3 supplementation for alleviating tumor-related pain. Similarly, clinical evidence of the effects of omega-3 supplementation in therapy-related pain is still scarce. Preclinical and clinical evidence on the neuroprotective effects of omega-3 PUFAs on chemotherapy-associated pain is provided in Table 1. Supporting the favorable analgesic actions of omega-3 PUFAs, a systematic review demonstrated that a nutritional supplement enriched with fish oil decreased the symptoms of fatigue and pain in patients during chemo- and/or radiotherapy, probably due to weight maintenance and reduced inflammatory status [93,94]. 

One might dispute the mechanisms underlying the analgesic effects of omega-3 PUFAs in cancer patients. A study, conducted by our research group, demonstrated that an omega-3 PUFA-enriched diet evoked analgesic effects in a mouse model of cyclophosphamide-induced visceral pain due to the overexpression of the FFA1 receptor in the spinal cord [11]. According to studies on other pain models, the activation of the FFA1 receptor induces the release of β-endorphin, noradrenaline, and serotonin, accounting for the analgesic actions of DHA [12,62]. Recently, it was observed that RvD2 decreased cancer pain in an experimental model of oral squamous cell carcinoma, probably via the downregulation of RvD2 receptors in this cancer cell, indicating that the resolution pathways could be suppressed. Another possible mechanism is the inhibition of several members of the transient receptor (TRP) family, such as TRPV1, TRPA1, TRPV3, and TRPV4 by RvD2 [95]. In virtue of the conversion of omega-3 PUFAs in specialized pro-resolution mediations, such as resolvins, protectins, and maresins, it is tempting to suppose that omega-3 PUFA supplementation prior to or during cancer treatment could inhibit or delay the appearance of treatment complications, such as pain and neuropathy.

## 5. Anorexia-Cachexia Syndrome

The use of omega-3 PUFA supplementation to treat anorexia-cachexia syndrome is commonly employed in cancer patients. However, the beneficial effects of these molecules for this complication are still questionable [101]. Particularly, there are no treatment plans for anorexia-cachexia syndrome in virtue of the multifactorial characteristics of this syndrome [102], demonstrating that an open discussion on the benefits of low-cost management, such as fish oil supplementation, is extremely important for clinical practice.

Regarding clinical evidence, studies using fish oil supplements, as a source of omega-3 fatty acids, demonstrated distinct effects on the development of cancer cachexia. For instance, head and neck cancer patients receiving an omega-3-enriched nutritional supplement received no benefits concerning cachexia features [103]. However, fish oil supplementation stabilized the weight of gastrointestinal cancer patients [104,105]. Interestingly, a recent systematic review evaluated the effects of fish oil for cachexia in advanced cancer, concluding that clinical evidence is still uncertain due to a weak methodology and a large variation of fish oil dosages. However, fish oil supplementation could benefit postoperative recovery and reduce complications, such as impaired wound healing and infections [106].

Remarkably, the plasmatic contents of omega-6/omega-3 and ALA/EPA were associated with muscle atrophy in cachectic cancer patients, indicating that these molecules may participate in the development of cancer cachexia [107,108]. A recent review article demonstrated that, during the last 23 years, in 31 clinical trials, cancer patients had some benefit from the use of omega-3 supplementation, mainly EPA. Regardless of the quantity of clinical and preclinical studies, this review concluded that the mechanisms underlying the benefits of omega-3 supplementation for cancer cachexia are still unknown [109]. Notably, EPA supplementation improved body weight and lean body mass in cancer patients by modulating circulating inflammatory markers, such as tumor necrosis factor (TNF), interleukin-1 β (IL-1β), interleukin-6 (IL-6), and interferon- γ (IFN-γ), demonstrating an inhibitory effect on inflammatory parameters related to muscle atrophy and lipolysis [110]. Additionally, Pappalardo et al. (2015) reviewed the issue of EPA as an anti-inflammatory agent, concluding that EPA supplementation has a positive effect in stabilizing lean body mass when compared to standard supplementation by diminishing the levels of C-reactive protein, IL-6, and TNF [111]. Two systematic reviews drew different conclusions regarding omega-3 supplementation. Colomer et al. (2007) demonstrated grade-B evidence (reasonable scientific evidence suggesting that the clinical benefits overcome the potential risks), suggesting that a dose of at least 1.5 g per day of EPA/DHA is related to enhanced clinical, biological, and quality of life parameters [112]. On the other hand, Mazzotta and Jeney (2008) showed that EPA and DHA failed to show significant clinical benefits related to body weight, lean body mass, survival, and life quality [113]. Concerning DHA alone, there is no clinical evidence demonstrating the effects of this molecule in cancer-associated cachexia.

Preclinical evidence provides further knowledge about the favorable effects of omega-3 supplementation in cancer-associated cachexia. In an in vivo model of cancer cachexia, EPA supplementation decreased the expression of zinc-α2-glycoprotein (ZAG), a lipolytic factor, in white and brown adipose tissue [114], demonstrating another mechanism of action related to EPA supplementation in cachexia. Regarding the EPA anti-lipolytic effect, Du et al. (2015) observed similar effects in the S180 ascitic cancer model after treatment with EPA derived from the starfish, *Asterias amurensis*, which was due to a reduction of ZAG, adipose triglyceride lipase (ATGL), hormone-sensitive lipase (HSL), peroxisome proliferator-activated receptor gamma coactivator 1-α (PGC-1α), and mitochondrial uncoupling protein 2 (UCP-2) expressions [115]. Notably, EPA supplementation alone reversed some aspects of the Lewis lung cancer-cachexia mouse model. However, training exercise, combined with EPA, promoted a stronger recovery after the development of lung cancer-associated cachexia in mice, mainly via the inhibition of the ubiquitin-proteasome system [116]. Concerning the effects of DHA alone in preclinical models of cancer-associated cachexia, the existing evidence is still scarce. In a mouse model of chemotherapy-induced body weight loss, a DHA-enriched diet was able to prevent body weight loss and to reduce glycerol release, indicating that DHA also had an anti-lipolytic effect [117]. In reference to the use of ALA in cancer cachexia, one study evaluated the effect of this molecule in a rat model of cancer cachexia using Oro Inca Oil (derived from *Sacha inchi* oil, a plant from the Andes), which is rich in ALA. Tumor-bearing rats receiving Oro Inca oil displayed an improvement of body weight, diminished IL-6, and TNF circulating levels, as well as decreased triacylglyceride (TAG) levels, demonstrating that ALA-rich oil also has anti-cachectic effects [118]. For a better comprehension of the scenery regarding cachexia management, Table 2 summarizes the data discussed above regarding the use of omega-3 PUFAs in this syndrome.

In light of the literature data, the use of omega-3 supplementation likely represents an interesting treatment option for cancer cachexia management in virtue of its anti-inflammatory, anti-lipolytic, and anti-catabolic actions. Since there are no studies involving cancer-associated cachexia and pro-resolution mediators, it is reasonable to propose that omega-3 conversion to pro-resolution mediators underlie part of the beneficial effects of omega 3 PUFAs in cancer. However, the absence of studies analyzing the role of free fatty acid receptors in cancer-induced cachexia limits our knowledge of the beneficial mechanisms mediating the omega-3 effects on this cancer-associated complication, indicating that novel studies are still required.

## 6. Major Depression Disorder (MDD)

Depression commonly occurs in cancer patients, affecting between 5% and 60% of oncological patients [121]. Pro-inflammatory cytokines, such as TNF, IL-1β, and IFN-γ, are released by the tumor–host interaction and can reach the hypothalamus, inducing a depression-like behavior. These cytokines can also stimulate the expression of serotonin and noradrenaline uptake transporters, leading to a diminished quantity of these neurotransmitters in the central nervous system [122]. Additionally, cancer-related depression can be evoked by cancer-independent mechanisms due to the impact of cancer diagnosis and stress [123]. Another probable mechanism, responsible for the development of cancer-related depression, is the upregulation of the leptin receptor, as observed in the gastric tissue of depressive gastric cancer patients, demonstrating that leptin may be involved in the pathogenesis of cancer-associated depression [124].

Regarding the participation of omega-3 in cancer-associated depression, it was observed that, in newly diagnosed Japanese lung cancer patients, the ALA and total omega-3 consumption was inversely associated with the development of depression. Nonetheless, EPA and DHA intake displayed no interaction whatsoever [125]. On the contrary, in the same population, a higher serum DHA was correlated with minor depression, but the authors indicated that the study had several limitations that could influence this conclusion [126]. Surprisingly, no further clinical trial investigated whether omega-3 PUFA supplementation could, to some extent, benefit cancer-related depression.

An overall analysis of the literature regarding the effects of omega-3 PUFAs in depression revealed controversial data. For instance, a meta-analysis study suggested that omega-3 supplementation is beneficial for depressed individuals [127]. On the other hand, Appleton et al. (2016) claimed that there is insufficient evidence to determine that omega-3 supplementation could be useful for depression treatment [128]. Nonetheless, Smith et al. (2017) demonstrated that a low-dose of DHA reduced depression and, interestingly, decreased insomnia, leading to a much-elevated quality of life [129]. In a cross-sectional analysis, a moderate dietary intake of omega-3 PUFAs was associated with a lower prevalence of depression [130]. It was noteworthy that patients with schizophrenia and depression displayed a low erythrocyte omega-3 index [131]. Similarly, Bigornia et al. (2016) demonstrated that the erythrocyte omega-3 index was inversely associated with depression in patients with elevated oxidative stress biomarkers [127]. Additionally, Müller et al. (2015) stated that a lack of omega-3 PUFAs in the brain could lead to a higher probability of developing depression and anxiety disorders [128]. Therefore, these studies support the hypothesis that omega-3 PUFAs can at least play a partial role in brain diseases, such as MDD.

As for the depression-like behavior related to cancer treatment, Orchard et al. (2016) considered that omega-3 supplementation could be beneficial for chemotherapy-induced cognitive alterations, such as depression [132]. Regarding preclinical evidence, rats submitted to repeated LPS administration displayed depressive-like behaviors, associated with decreased levels of monoamines besides an increase of apoptotic markers in the hippocampus and prefrontal cortex. It was noteworthy that fish oil supplementation reversed all of these effects, displaying an important anti-inflammatory action [133]. Fat-1 is a transgenic mouse model that endogenously transforms omega-6 to omega-3 by expressing *C. elegans fat-1* gene-encoding omega-3 desaturase [134]. Strikingly, these animals displayed benefits regarding neuroinflammation and oxidative stress in the depression model induced by LPS. Interestingly, this study showed that LPS depression induces a pro-inflammatory M1 phenotype in hyperactive microglia, and endogenous omega-3 shifted this phenotype to an anti-inflammatory phenotype M2 [135].

According to Larrieu and Layé (2018), omega-3 PUFAs exhibit neuroprotective effects in the development of brain diseases, such as depression and anxiety, particularly by the sensing activity of free fatty acid receptors [136]. The effects, modulated by an activation of the free fatty acid receptors, mainly FFA1, were only observed in experimental models, but it is possible to surmise that the activation of this receptor might be important for depression management. For instance, the repeated administration of GW9508, an FFA1 receptor agonist, was able to diminish the immobility time of mice during a forced swimming test. Additionally, the same study showed that DHA and AA levels were decreased in the hippocampus after the behavioral test, although the FFA1 expression remained unaltered [137]. Corroborating these data, Aizawa et al. (2016) demonstrated that FFA1 knockout mice exhibited augmented anhedonia and altered levels of serotonin, dopamine, and noradrenaline in the hippocampus [138]. Similar effects were observed in FFA1 knockout female mice. In addition to the increased anhedonia, female mice presented reduced maternal care, such as negligence and infanticide; reduced locomotor activity; and decreased social interaction [139]. Concerning the effects of pro-resolution mediators in depression, molecules derived from DHA were further investigated rather than EPA-derived molecules, revealing the antidepressant effects of resolvins of the D series when given i.c.v. in rodent models [47,140,141,142].

Taking into account the effects promoted by omega-3 PUFAs in different animal models of depression and depressed individuals, as summarized in Table 3, it is possible to assume that omega-3 and omega-3-derived mediators can play an important role in MDD, possibly via the activation of FFA1 or resolution pathways. Considering the evidence mentioned above, the management of depression in cancer patients could very well be another indication supporting omega-3 PUFA supplementation in the treatment of cancer. An improvement of the depression symptoms by omega 3 or related fatty acid dosing might contribute to a better response to cancer treatment and an overall life quality improvement of the affected individuals.

## 7. Paraneoplastic Syndromes

Paraneoplastic syndromes are multiple clinical complications that are related to tumor metabolites, but they are considered rare. These syndromes are categorized as neurological, endocrinological, hematological, dermatological, and rheumatological complications [26]. Interestingly, it is possible to suppose that complications, such as anorexia-cachexia syndrome and cancer pain, could also be classified as paraneoplastic syndromes due to their pathophysiology mechanism.

Endocrine paraneoplastic syndromes occur due to the interaction of substances released by the tumor cells originating from endocrine or neuroendocrine cells, which are distributed through different parts of the human body. Non-endocrine tumor cells can also liberate substances, promoting similar symptoms and sharing the same clinical features [27]. The most common endocrine paraneoplastic syndromes are hypercalcemia, the syndrome of inappropriate antidiuretic hormone secretion (SIADH), and Cushing’s syndrome. On the other hand, other complications, such as non-islet cell tumor hypoglycemia, gynecomastia, acromegaly, hypertension, ovarian hyperstimulation syndrome, hyperprolactinemia, hyperthyroidism, and secretory diarrhea, are considered rare endocrine paraneoplastic syndromes [145]. As for hypercalcemia, this syndrome develops due to the protein related to the parathyroid hormone-related peptide (PTHrP) released by the tumor, stimulating bone resorption and leading to higher levels of serum parathyroid hormone (PTH) and osteoclast hyperactivity. Interestingly, the same PTHrP secretion induces the browning of the adipose tissue, leading to an increase of energy expenditure and leading to the cachectic state [146]. One might presume that omega-3 supplementation could be beneficial for hypercalcemia-associated bone resorption because it was observed that, in an animal model of apical periodontitis, omega-3 supplementation reduced bone resorption by the downregulation of the inflammatory cells influx [147]. Moreover, the combination of omega-3 supplementation and exercise in postmenopausal healthy women promoted diminished serum PTH levels, leading to an improvement of skeletal health [148]. Nevertheless, there is no evidence linking paraneoplastic tumor-induced hypercalcemia and omega-3, as well as in relation to other kinds of paraneoplastic endocrine syndromes.

Neurological paraneoplastic syndromes are a consequence of the production of tumor antibodies, known as *onconeural antibodies*, which react with the nervous system, promoting damage [149]. Interestingly, neurological paraneoplastic syndromes are detected before cancer diagnosis and can help patient prognosis by a premature treatment initiation. The most common neurological paraneoplastic syndromes are encephalopathies, neuropathies, encephalomyelitis, cerebellar degeneration, myelitis, myasthenic syndrome, myasthenia gravis, neuromyotonia, dermatomyositis, and stiff person syndrome [149,150]. Limbic encephalitis is one of the most common paraneoplastic syndromes and develops by a reaction to Anti-Hu (HuD antigen; small cell lung carcinoma), Anti-Ma2 (Ma proteins; germ-cell tumors), or Anti-NMDA (N-methyl-D-aspartate; teratomas), which are mainly characterized by neuroinflammation. In relation to general encephalitis, omega-3 supplementation demonstrated neuroprotective effects against traumatic brain injury by reducing microglial activation and regulating the Toll-like receptor 4 (TLR4)/NF-kB signaling pathway [151]. Again, there is no evidence linking the use of omega-3 supplementation and neurological paraneoplastic syndromes, but it is possible to presume that omega-3 could be beneficial in some kinds of neuropathy. This notion is based on the available literature related to omega-3 supplementation and other types of neuropathy. In a mouse model of diabetic neuropathy, fish oil supplementation, and daily systemic administration of RvE1 and RvD1 reversed thermal hypoalgesia, mechanical allodynia, reduced motor, and sensory nerve conduction and decreased the innervation of cornea and skin [152]. Similar effects were observed by Yorek et al. (2016) in a mouse model of diabetic neuropathy, when fish oil and RvD1 promoted benefits related to the development of neuropathy but not to diabetes itself [153]. Interestingly, this last study demonstrated that the combination with fish oil and salsalate promoted similar anti-inflammatory effects when compared to RvD1 or fish oil alone, probably due to the acetylation of cyclooxygenases, resulting in an increase of RvD1 and leading to the resolution of the inflammatory process.

Rheumatologic paraneoplastic syndromes develop in a similar way to endocrine paraneoplastic syndromes [154]. This kind of paraneoplastic syndrome is also considered rare, and it can appear two years before cancer diagnosis. The most common rheumatological paraneoplastic syndromes are hypertrophic osteoarthropathy, polyarthritis, tumor-induced osteomalacia, and cancer-associated myositis [155]. As for the other paraneoplastic syndromes, there is no evidence concerning the effects of omega-3 supplementation in these alterations. Therefore, it is only possible to assume that omega-3 could be beneficial based on the existing literature of similar alterations. In dogs suffering from osteoarthritis, fish oil supplementation attenuated oxidative stress, and inflammatory markers after the dietary intervention, but no pain assessment was evaluated [156]. Additionally, DHA supplementation in rheumatoid arthritis patients significantly reduced the clinical and biochemical symptoms of inflammation [157]. Regarding cancer-associated myositis, an in vitro study evaluated the effects of DHA in LPS-induced inflammation in myoblast cells (C2C12 myotubes), and it was observed that 30 mM of DHA prevented lipotoxicity and skeletal muscle inflammation [158]. Tumor-induced osteomalacia is caused by tumors that secrete fibroblast growth factor 23 (FGF23), inducing hypophosphatemia, leading to a reduced osteoblast differentiation and matrix mineralization [159]. Literature related to tumor-induced osteomalacia and omega-3 fatty acids is nonexistent. Nevertheless, in renal transplant patients, EPA/DHA intake decreased FGF23 circulating levels [160]. Thus, omega-3 supplementation might control tumor-associated hypophosphatemia, consequently reducing the development of osteomalacia.

Regarding dermatological paraneoplastic syndromes, they can represent 1% of the first diagnostic in cancer patients. Skin alterations related to neoplasms are caused by vascular alterations or a high differentiation of keratinocytes/fibroblasts. The more rapidly the skin manifestation appears, the higher the probability that it will be associated with cancer [161]. Commonly, paraneoplastic dermatological syndromes are acanthosis nigricans, dermatomyositis, erythroderma, leukocytoclastic vasculitis, paraneoplastic pemphigus, polymyalgia rheumatica, and Sweet’s syndrome. Concerning the skin paraneoplastic alterations, there is no evidence regarding the effects of omega-3 supplementation, even though the importance of omega-3 dietary supplementation for skin health is well-known [162]. Moreover, concerning wound-healing effects, omega-3 supplementation promotes beneficial therapeutic effects in healthy subjects [163].

Finally, hematological paraneoplastic syndromes are rarely symptomatic and usually related to the presence of a tumor. The most common hematological paraneoplastic syndromes are eosinophilia, granulocytosis, pure red cell aplasia, and thrombocytosis. Generally, hematological alterations are often induced by an increase of pro-inflammatory cytokine circulation. In dogs suffering from osteoarthritis, fish oil reduced circulating basophils and monocytes, but it failed to prevent lymphocyte, neutrophil, and eosinophil alterations [156]. Considering the role of omega-3 pro-resolution mediators, resolvins and protectins promoted the reduction of circulating eosinophils [164].

There is limited preclinical or clinical evidence on the effects of omega-3 supplementation in cancer patients that develop paraneoplastic syndromes. However, it may be possible to presume that omega-3 supplementation could be beneficial—or, in any case, harmless—for cancer patients with paraneoplastic syndromes and that a higher daily intake of omega-3 fatty acids could prevent the evolution of future paraneoplastic alterations. Finally, the lack of evidence regarding omega-3 supplementation effects on paraneoplastic syndromes indicates the need for future cancer-associated complications research.

## 8. Literature Trends Regarding Omega-3 PUFAs and Cancer Complications

With the aim of visualizing the existing evidence discussed in this review, we used the VOSViewer software (Leiden University, Netherlands) to build a co-occurrence network of terms, based on publications retrieved from PubMed, using the following keywords: “cancer pain”, “cancer depression”, “cancer cachexia”, and “paraneoplastic syndrome”, each combined with “omega-3”. From the database, containing data of about 297 articles (original and review articles), VOSViewer extracted the most relevant and repeated terms in the retrieved publications (considering title, keywords, and abstract). To be considered, a term had to appear in at least 10 articles.

**Figure 1:**
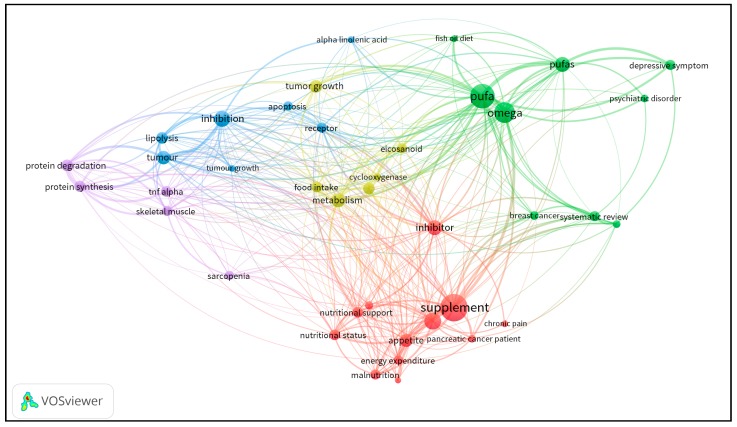
A co-occurrence network of terms based on publication data retrieved from PubMed using keywords that reproduce the search performed for this review article. The circle sizes represent the number of articles featuring the corresponding term; the links between the circles represent the co-occurrence of terms in the same articles; and the line thickness is dependent on the number of articles sharing the terms. Different colors identify clusters composed by closely related terms.

As can be seen in Figure 1, the circles represent terms as they appear in the database, and their size is related to the number of articles in which they appear. A line connecting two terms indicates that both terms occur in the same article, and the thicker the lines, the larger the number of articles sharing both terms, which can be considered a measure of the association strength between the terms. The distribution of the circles in the network is also related to the co-occurrence so that the circles are positioned close to others, depending on the association strength between the terms they represent.

The network also represents clusters of terms, depicted in different colors. Such clusters allow for an understanding of the structure of the publications set because they result from a process that considers the similarity of the co-occurrences of the terms. It is possible to observe that the main clusters are green and red. The green ones comprise terms related to omega-3 PUFAs and their use in diseases, such as cancer and mental disorders. Curiously, the term “systematic review” appears in the same cluster, demonstrating that there are more reviews on these subjects. The red cluster comprises terms related to “nutritional therapy” in cancer and other complications, such as “chronic pain”. Importantly, the word “inhibitor” appears in red but is more distant from the red cluster core, probably due to its co-occurrence with terms in other clusters, although it is related to “supplementation” and “nutritional support”. The third major cluster is in blue, and it is directly related to pharmacological and molecular terms, such as “tumour growth” and “tumour”. Additionally, ALA appears in the same cluster, demonstrating that the use of ALA can be related to the “inhibition” of molecular pathways. Near these terms, one can observe that “tumour growth” is in yellow. Despite being located near to the blue cluster, due to its close relation to other terms that are also linked to the British form of writing “tumour”, it belongs to the yellow cluster, which is positioned in a more central area. The location of the yellow cluster results from the co-occurrence of its terms with many others belonging to other clusters. The fourth cluster is in purple, and its terms are linked to protein and muscle metabolism. Moreover, this type of analysis demonstrates the problem of standardizing some terms throughout the scientific literature, as it can be seen in “pufa” and “pufas”, emerging as two different terms. The organization of the existing literature data in such a co-occurrence network clearly shows the distance between cancer complications, such as “chronic pain” (red cluster), “depressive symptom” (green cluster), and “protein degradation” (purple cluster), which demonstrate that there is a lack of publications addressing the relationship between omega-3 PUFAs and the cancer-related complications revised in this review article.

## 9. Conclusions

Since the 1970s, omega-3 PUFAs have been a subject of multiple investigations due to their ability to suppress inflammatory processes. In recent years, it has become possible to identify some of the mechanisms of action of these molecules besides the potential molecular targets. As can be seen throughout this critical review, an omega-3 supplementation is widely employed in cancer patients, mainly as an adjunctive treatment. The identification of pro-resolution mediators derived from omega-3 fatty acids opened up a variety of therapeutic possibilities for different pathologies. More recently, the identification of free fatty acid receptors as therapeutic targets for omega-3 PUFAs also revealed a plethora of beneficial opportunities. Importantly, it is imperative to emphasize that, in 2018, Omegaven^®^ (Fresenius Kabi, Germany) was approved by the Federal Drug Administration (FDA) for parenteral nutrition in cholestasis, demonstrating that it is important to consider omega-3 fatty acids as a therapeutic option that is related to all regulatory functions, similar to a new pharmaceutical drug [165].

**Figure 2:**
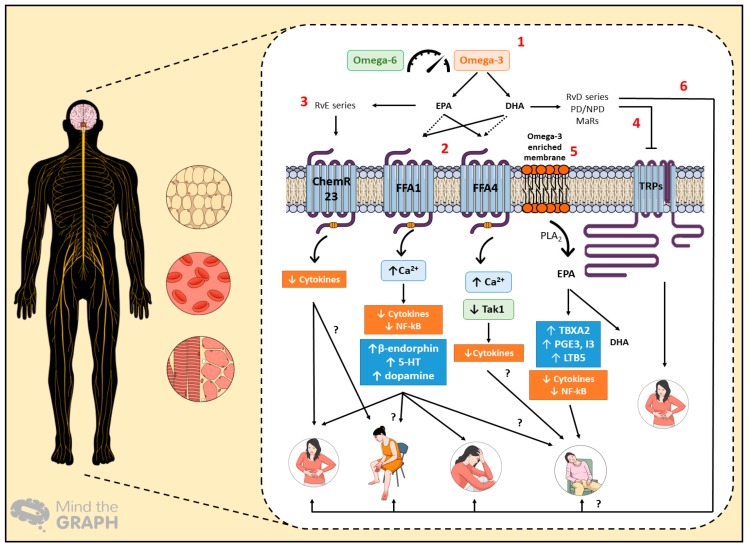
The proposed mechanisms of action for omega-3 polyunsaturated fatty acid (PUFA) intake in cancer-related complications, which affect the central and peripheral nervous system, besides adipose tissue and skeletal muscle. Hematological changes depict the switched production of systemic inflammatory mediators under cancer progression. (1) The balance between omega-3 and omega-6 PUFAs is essential for the generation of pro-resolution mediators, the sensing of free fatty acid receptors, and membrane modulation. (2) Eicosapentaenoic acid (EPA) and docosahexaenoic acid (DHA) can stimulate Free Fatty Acid Receptor 1 (FFA1) and Free Fatty Acid Receptor 4 (FFA4) receptors in different ways, leading to anti-inflammatory effects. FFA1 activation can evoke analgesia and antidepressant effects. Additionally, this receptor could have benefits in relation to paraneoplastic syndromes, such as neuropathy and cachexia-anorexia syndrome, represented herein by fatigue. (3) E-series resolvins (RvE) promotes anti-inflammatory effects via ChemR23 activation, likely contributing to the alleviation of painful symptoms. They might also induce beneficial effects in other paraneoplastic syndromes. (4) The inhibitory effects of D-series resolvins (RvD) on transient receptor (TRP) channels could also produce favorable effects on cancer-related pain. (5) As for the omega-3 PUFA membrane enrichment, the production of thromboxane A2 (TBXA2), prostaglandins E3 and I3 (PGE3/I3), and leukotriene B5 (LTB5) promotes anti-inflammatory effects that prompt cachectic patients’ welfare. (6) Finally, RvD-series, protectins/neuroprotectins (PD/NPD), and maresins (MaRs) have beneficial effects on pain and depression; thus, they could similarly relieve the signs and symptoms of paraneoplastic and cachexia-anorexia syndromes.

Regarding the beneficial effects of omega-3 fatty acids in cancer-related complications, additional studies are still needed, mainly randomized clinical trials with omega-3 supplementation, due to the deficiency of clinical literature evidence. There is also a lack of scientific evidence regarding whether omega-3 PUFAs are able to significantly prevent or address cancer-related complications, depending on the cancer stage, from dysplasia to carcinoma and metastasis. In Figure 2, we attempted to summarize the possible pathways by which omega-3 PUFAs might promote favorable effects in cancer-related complications, such as pain, paraneoplastic syndrome, depression, and cachexia-anorexia syndrome. The definition of these mechanisms might also account for the development of novel strategies based on omega-3 PUFAs, contributing to an improvement of the life quality of cancer patients in the near future.

## Figures and Tables

**Table 1:** A summary of the articles discussed above regarding the effects of omega-3 PUFAs in cancer and cancer-treatment complications.

Authors	Cancer-Related Complication	Species	Cancer Type	Treatment Scheme	Major Outcome
Hershmann et al., 2015 [96]	Aromatase-inhibitor associated arthralgia	Human	Breast cancer	3.3 g ^1^ FO (560 mg EPA + DHA; 40:20)	Decreased pain, evaluated by the ^2^ BPI between the baseline and week 24 (*p* < 0.01)
Shen et al., 2018 [97]	Aromatase-inhibitor associated arthralgia	Human	Breast cancer (obese)	3.3 g FO (560 mg EPA + DHA; 40:20)	Pain reduction in ^3^ BMI > 30 kg/m² patients (*p* = 0.02)
Martínez et al., 2018 [98]	Aromatase-inhibitor musculoskeletal symptoms (AIMSS)	Human	Breast cancer	460 mg EPA + DHA 12.5 mg hydroxytyrosol 50 g curcumin	Decrease of the BPI total score after 30 days (*p* = 0.011)
Ghroreishi et al., 2012 [99]	Paclitaxel-induced neuropathy	Human	Breast cancer	640 mg FO (54% DHA + 10% EPA)	70% did not develop neuropathy no pain score assessed
Maschio et al., 2018 [100]	Bortezomib-related neuropathy	Human	Multiple myeloma	Neuronorm^®^ (400 mg DHA + 600 mg ALA)	Pain failed to increase significantly (*p* = 0.33)
Freitas et al., 2016 [11]	Cyclophosphamide-induced hemorrhagic cystitis	Mice	-	20% FO-enriched diet or 1 µmol/kg i.p.	Decrease in spontaneous pain behavior and abdominal allodynia (*p* < 0.01)
Ye et al., 2018 [95]	Oral and paw cancer pain	Mice	Oral squamous cell carcinoma	RvD1 (100 ng or 200 ng) or RvD2 (100 ng or 200 ng) i.p.	RvD2 inhibited thermal and mechanical pain; RvD1 inhibited thermal pain

^1^ FO: Fish oil; ^2^ BPI: Brief pain inventory; ^3^ BMI: body mass index.

**Table 2:** A brief summary of the selected articles on the use of omega-3 PUFAs as a treatment for cancer-related cachexia.

Authors	Cancer-Related Complication	Species	Cancer Type	Treatment Scheme	Major Outcome
Hanai et al., 2018 [103]	Cachexia-anorexia syndrome	Human	Head and neck squamous cell carcinoma	Prosure^®^ (1056 mg EPA)	No significant difference among experimental groups
Persson et al., 2005 [104]	Cachexia-anorexia syndrome	Human	Advanced gastrointestinal cancer	30 mL/d ^1^ FO (4.9g EPA + 3.2 g DHA)	FO stabilized weight in 27% patients
Shirai et al., 2017 [105]	Cachexia-anorexia syndrome	Human	Advanced gastrointestinal cancer	Prosure^®^ (1.1 g EPA + 0.5 g DHA)	Increase of body weight and lean body mass (*p* = 0.002/*p* < 0.001)
Werner et al., 2017 [119]	Cachexia-anorexia syndrome	Human	Pancreatic cancer	6.9 g EPA/13.6 g DHA in 100 g or 8.5 g EPA/ 12.3 g DHA in 100g	No significant differences between omega-3 PUFA treatments
Solis-Martínez et al., 2018 [110]	Cachexia-anorexia syndrome	Human	Head and neck squamous cell carcinoma	2 g EPA	Weight and ^2^ LBM maintenance
Hajjaji et al., 2012 [117]	Chemotherapy-induced cachexia	Rat	Chemically-induced tumor + doxorubicin treatment	DHA-enriched diet (80 g/kg diet)	DHA diet avoided weight loss
Schissel et al., 2015 [118]	Cancer-associated cachexia	Rat	Breast carcinoma (Walker 256 cell line)	53.6% EPA + DHA or 54.4% ALA	ALA and EPA improved weight gain (cachectic vs. cachectic + omega-3 *p* < 0.05)
Du et al., 2015 [115]	Cancer-related cachexia	Mice	Sarcoma (S180 cell line)	42% EPA + 6.8% DHA	Decreased lipolysis and increased body weight (*p* < 0.001)
Penna et al., 2011 [116]	Cancer-related cachexia	Mice	Lewis lung carcinoma	EPA (0.5 g/kg) or EPA (0.5 g/kg) + exercise	EPA + exercise significantly improved muscle weight (*p* < 0.05)
Muzio et al., 2016 [120]	Cachexia in vitro model	Human	Lung adenocarcinoma	50 µM EPA + DHA	Myoblast formation

^1^ FO: Fish oil; ^2^ LBM: lean body mass.

**Table 3:** A brief summary of the selected articles using omega-3 PUFAs as treatment for depression.

Authors	Clinical or Experimental Condition	Species	Treatment Scheme	Major Outcome
Chhetry et al, 2016 [143]	MDD	Human	4 g ^1^ FO (1.6 g EPA + 0.8 g DHA)	Improved MDD-related white matter deficiency
Smith et al., 2017 [129]	MDD	Human	260 mg or 520 mg DHA	54% of patients showed a reduction of depression severity ≥ 50%
Wu et al., 2018 [144]	Chemotherapy-induced depression	Rat	1.5 g/kg omega-3 PUFAs (34% EPA + 24% DHA)	PUFAs inhibited depressive-like behaviors (*p* < 0.001)
Dang et al., 2018 [133]	LPS-induced depression	Rat	1.5 g/kg omega-3 PUFAs (34% EPA + 24% DHA)	Omega-3 PUFAs decreased depressive behavior (*p* < 0.001)
Nishinaka et al., 2014 [137]	Behavioral despair paradigm	Mice	GW9508 (1.0, 10 or 25 µg/mouse) i.c.v	FFA1 activation decreased immobility in a tail suspension test (*p* < 0.05)
Deyama et al., 2017 [140]	LPS-induced depression	Mice	RvD1 (10 ng i.c.v.) or RvD2 (10 ng i.c.v.)	Both treatments inhibited depressive-like behaviors (*p* < 0.005)
Deyama et al., 2018 [141]	LPS-induced depression	Mice	RvE3 (10 and 100 ng i.c.v.)	Inhibition of depressive behavior (*p* < 0.005)
Ishikawa et al., 2017 [142]	Chronic unpredictable stress-related depression	Mice	RvD1 (10 ng i.c.v.) or RvD2 (10 ng i.c.v.)	Both treatments inhibited depressive behavior for 24 h (*p* < 0.05)

^1^ FO: Fish oil.
